# Therapeutic Potentials of Inhibition of Jumonji C Domain-containing Demethylases in Acute Myeloid Leukemia

**DOI:** 10.4274/tjh.galenos.2019.2019.0083

**Published:** 2020-02-20

**Authors:** Duygu Koca, Nurcan Hastar, Selin Engür, Yağmur Kiraz, Gizem Tuğçe Ulu, Demet Çekdemir, Yusuf Baran

**Affiliations:** 1 İzmir Institute of Technology, Department of Molecular Biology and Genetics, İzmir, Turkey; 2 Anadolu University Faculty of Pharmacy, Department of Pharmacology, Eskişehir, Turkey; 3 Sakarya University Faculty of Medicine, Department of Hematology, Sakarya, Turkey

**Keywords:** Acute myeloid leukemia, Methylstat, Jumonji C domain, Histone methylation

## Abstract

**Objective:**

Acute myeloid leukemia (AML) is a complex disease affected by both genetic and epigenetic factors. Histone methylation and demethylation are types of epigenetic modification in chromatin remodeling and gene expression. Abnormal expression of histone demethylases is indicated in many types of cancer including AML. Although many commercial drugs are available to treat AML, an absolute cure has not been discovered yet. However, inhibition of demethylases could be a potential cure for AML. Methylstat is a chemical agent that inhibits the Jumonji C domain-containing demethylases.

**Materials and Methods:**

The cytotoxic and apoptotic effects of methylstat and doxorubicin on HL-60 cells were detected by MTT cell viability assay, double staining of treated cells with annexin-V/propidium iodide, and caspase-3 activity assay. Mitochondrial activity was analyzed using JC-1 dye. The expression levels of the *BCL2* and *BCL2L1* anti-apoptotic genes in HL-60 cells were determined using real-time polymerase chain reaction (PCR). Lastly, the cytostatic effect was determined by cell cycle analysis.

**Results:**

In our research, cytotoxic, cytostatic, and apoptotic effects of methylstat on human HL-60 cells were investigated. Cytotoxic and cytostatic analyses revealed that methylstat decreased cell proliferation in a dose-dependent cytotoxic manner and arrested HL-60 cells in the G2/M and S phases. Methylstat also induced apoptosis through the loss of mitochondrial membrane potential and increases in caspase-3 enzyme activity. The expression levels of *BCL2* and *BCL2L1* were also decreased according to real-time PCR results. Finally, the combination of methylstat with doxorubicin resulted in synergistic cytotoxic effects on HL-60 cells.

**Conclusion:**

Taken together, these results demonstrate that methylstat may be a powerful candidate as a drug component of AML treatment protocols.

## Abstract (tr)

**Amaç:**

Akut myeloid lösemi (AML) hem genetik hem de epigenetik faktörlerden etkilenen kompleks bir hastalıktır. Histon metilasyonu ve demetilasyonu, kromatin yeniden yapılanması ve gen ekspresyonu tayinindeki önemli epigenetik modifikasyonlarındandır. Histon demetilazların anormal ekspresyonu, AML dahil birçok kanser türünde etkilidir. AML’yi tedavi etmek için birçok ticari ilaç mevcut olmasına rağmen, kesin tedavisi bulunmamaktadır. Bu nedenle, demetilazların inhibisyonu AML için potansiyel bir tedavi olabilir. Methylstat, demetilaz içeren Jumonji C domain-inhibe eden bir kimyasal maddedir.

**Gereç ve Yöntemler:**

Methylstat ve doksorubisinin HL-60 hücreleri üzerindeki sitotoksik ve apoptotik etkisi, sırasıyla MTT canlılık testi, annexin-V/propidium iyodür çift boyaması ve kaspaz-3 aktivite testi ile saptandı. Ayrıca, hücrelerdeki mitokondriyal aktivite JC-1 boyası kullanılarak analiz edildi. HL-60 hücrelerinde *BCL2* ve *BCL2L1* apoptotik olmayan genlerin ekspresyon seviyeleri, gerçek zamanlı polimeraz zincir reaksiyonu (PZR) kullanılarak belirlendi. Son olarak, sitostatik etki hücre döngüsü analizi ile belirlenmiştir.

**Bulgular:**

Araştırmamızda, methylstatın HL-60 hücreleri üzerindeki sitotoksik, sitostatik ve apoptotik etkileri olduğu belirlenmiştir. Sitotoksik ve sitostatik analizlerde methylstatın, HL-60 hücrelerinde doza bağlı hücre çoğalmasını azalttığını ve G2/M ve S fazlarındaki sitotoksik etkisinin arttığı ortaya koydu. Methylstat ayrıca, mitokondriyal membran potansiyelini düşürdüğü ve kaspaz-3 enzim aktivitesindeki artışlarla apoptozu indüklemiştir. Ayrıca, gerçek zamanlı PZR sonuçlarına göre *BCL2* ve *BCL2L1* genlerinin ekspresyon seviyeleri azalmaktadır. Son olarak, methylstatın doksorubisin ile kombinasyonu, HL-60 hücreleri üzerinde sinerjik sitotoksik etkilere yol açmıştır.

**Sonuç:**

Methylstatın AML için tam bir tedavi bulmak için bir ilaç bileşeni olarak güçlü bir aday olabileceğini göstermiştir.

## Introduction

Acute myeloid leukemia (AML) is a disease caused by the rapid proliferation of neoplastic cells [1]. AML is determined by the accumulation of blast cells and the blocking of the differentiation of myeloid cells in bone marrow. This abnormal proliferation leads to the disruption of bone marrow function and the maturation of white blood cells that are necessary for the immune system [2,3]. Treatment of AML is provided by cytotoxic drugs and bone marrow transplantation [4]. However, the exact treatment of AML is still unclear [5]. AML is known as a genetic disorder resulting from chromosomal translocations. However, developing depth genome sequencing shows that epigenetic factors and abnormalities also have a role in the progression of AML [6]. According to cytogenetic tests, ~50% of AML patients possess normal karyotypes [7]. These outcomes revealed that not only chromosomal alterations but also epigenetic abnormalities might have roles in the progression of AML [8]. Histone-modifying enzymes play crucial roles during the dynamic regulation of gene expression and cell identification [9]. As histone-modifying enzymes, histone demethylases have a potent role in the regulation of gene expression through modulation of histone methylation. Recently, overexpression of several histone demethylases has been observed in many types of cancer. Different drugs are developing to regulate DNA methylation, which is a critical point for targeted cancer therapy and decreasing drug resistance of cancer cells [10,11].

There are two families of histone demethylases. The LSD family has two subfamilies known as LSD1 (also known as KDM1A) and LSD2 (also known as KDM1B). These enzymes contain an amine oxidase-like domain and are flavin-dependent demethylases [12,13]. The second family of histone demethylases comprises the catalytic Jumonji C (JmjC) domain-containing demethylases. The enzymatic reaction mechanism of demethylases containing the JmjC domain requires two cofactors, Fe(II) and 2-oxoglutarate. The JmjC domain behaves differently in terms of its reaction mechanism. In contrast to LSD demethylases, JmjC achieves removal of trimethyl marks. The biochemical and biological functions of the JmjC domain are interesting for cancer treatment due to the regulation of chromatin remodeling and epigenetic factors that provide genome stability [14,15].

In recent studies, overexpression of several JmjC domain-containing histone demethylases (JHDMs) was determined for many types of cancer, including leukemia [16,17]. Therefore, JHDMs could be a therapeutic target for AML treatment. The compounds that inhibit JHDMs have potential as candidate anti-cancer agents [18]. In this study, we aimed to examine the cytotoxic, cytostatic, and apoptotic effects of methylstat, a selective inhibitor of a large set of JHDMs, on AML cells.

## Materials and Methods

### Cell Culture

Human HL-60 cells were obtained from the ATCC. The required medium was RPMI-1640, containing 10% fetal bovine serum and 1% penicillin and streptomycin. The cells were cultured in a CO_2_ incubator with adjusted conditions of 5% CO_2_ and 37 °C. Every 3 days, the cells were passaged and fresh medium was provided in order to properly maintain the cells.

### Reagents, Drugs, and Compound

Methylstat was dissolved in dimethyl sulfoxide (DMSO) and the final concentration of methylstat was 50 mM. Doxorubicin hydrochloride (injectable lyophilized powder form, Teva Pharmaceutical Industries, Pharmachemie BV) was kindly provided by Dr. Gökmen Sevindik from Dokuz Eylül University, İzmir, Turkey. It was prepared by dissolving powder in sterile molecular biology water and the final concentration was obtained as 3.4 mM. Required concentrations were calculated and necessary dilutions were carried out with complete medium.

### MTT Cell Viability Assay

3-[4.5-Dimethylthiazol-2-yl]-2.5-Diphenyltetrazolium bromide (MTT) is an auxiliary agent that gives a yellow color when dissolved in phosphate-buffered saline (PBS). The main stock solution was prepared in a concentration of 5 mg/mL. To sterilize the solution, it was filtered through a 0.45-µM filter inside the hood, and 1x10^4^ HL-60 cells were inoculated in wells of 96-well plates. Methylstat was diluted in determined concentrations and added to wells. After 72 h, 20 µL of MTT solution was added to each well. Thus, the ratio of MTT was adjusted to 1:10. The 96-well plates were incubated at 37 °C in an incubator with 5% CO_2_ for 3 h. At the end of incubation, plates were centrifuged at 1800 rpm for 10 min. Supernatants were removed by gentle tapping and 150 µL of DMSO was added as a solvent to dissolve the formazan salts. Plates were shaken on a shaker at 150 rpm for 5 min to totally dissolve all the crystals. The absorbance was measured at 570 nM by using a spectrophotometer (Thermo Multiskan Spectrum). This MTT assay was applied to HL-60 cells with doxorubicin with the same procedure. For MTT experiments, the following application doses were used: 50, 25, 12.5, 6.2, 3.1, 1.6, 0.8, 0.4, 0.2, 0.1, and 0.05 µM.

### Double Staining of Treated Cells with Annexin-V/Propidium Iodide (PI)

In healthy cells, phosphatidylserines (PSs) are located in the inner part of the membrane, while in apoptotic cells, the asymmetry of the cell membrane is destroyed and PSs face an extracellular matrix of cells. Annexin-V has high affinity to PSs. Thus, apoptosis can be detected with the help of the binding of annexin-V to PSs. On the other hand, while living cells are impermeable to propidium iodide (PI), membranes of dead cells are permeable to PI. Therefore, double staining provides us an understanding of whether the apoptosis is early or late [19]. HL-60 cells were grown in the absence and presence of methylstat and incubated at 37 °C and 5% CO_2_ for 72 h. The cells were then collected into 15-mL Falcon tubes and washed with PBS. To obtain pellets, tubes were centrifuged at 800 rpm for 5 min and the cells were washed with cold PBS two times. Obtained pellets were resuspended with 1X annexin binding buffer and transferred to properly labeled FACS tubes, and 5 µL of FITC and 5 µL of PI were added to each sample. Tubes were incubated for 15 min in the dark. At the end of incubation, 400 µL of 1X annexin binding buffer was added and the results were obtained by using flow cytometry (FACSCanto, BD, USA).

### Detection of Caspase-3 Activity

The caspase family, including cysteine-dependent, aspartate-specific proteinases, has a role in apoptosis. Therefore, the detection of caspase-3 activity is important in the determination of apoptotic signals [20]. Colorimetric determination of caspase-3 activity was determined with the help of a BioVision assay. In this assay, DEVD-pNA (chromophore *p*-nitroaniline) is the prepared substrate for the caspase-3 enzyme. When the caspase-3 enzyme cleaves pNA from DEVD by recognizing the DEVD sequence, pNA gives an emission in spectrophotometry. In our experiments, 1x10^6^ cells were seeded in 6-well plates and incubated in the absence or presence of increasing concentrations of methylstat. After 72 h of incubation at 37 °C in an incubator with 5% CO_2_, cells were collected in Falcon tubes and centrifuged at 1000 rpm for 10 min. Supernatants were taken into Eppendorf tubes and 100 µL of lysis buffer was added to each sample. They were incubated for 10 min on ice. Centrifugation was adjusted to 4 °C and the samples were centrifuged at 10,000 x g for 1 min. Supernatants were taken into other labeled Eppendorf tubes and 100 µL of lysis buffer was added. Halves of the supernatants were stored at -80 °C for the Bradford assay, and 50 µL of each sample was transferred to a 96-well plate. Dithiothreitol (DTT) was added to 2X reaction buffer (10 mM DTT in 50 µL of reaction buffer) and 5 µL of DEVD-pNA was added to each sample, and the plates were incubated at 37 °C with 5% CO_2_ in the dark. By using a spectrophotometer, the absorbance of samples was measured at 405 nM. These absorbance values were used to determine protein concentrations by standardization with Bradford assay absorbance.

### Determination of Loss of Mitochondrial Membrane Potential

Mitochondria play a crucial role during apoptosis. Cells lose their mitochondrial membrane potential (MMP). In order to detect this loss, a dye is utilized called 5,5′,6,6′-tetrachloro-1,1′,3,3′- tetraethyl benzimidazol carbocyanine iodide (JC-1). It is a cytofluorimetric, lipophilic, and cationic dye. In healthy cells, this dye aggregates in the mitochondria and gives a red fluorescence, whereas in apoptotic or dead cells, the dye cannot pass through mitochondria and stays in the cytoplasm in monomer form. This monomer form of the dye gives a green fluorescence [21]. Briefly, 1x10^6^ cells were seeded in 6-well plates and increasing concentrations of methylstat were added to the cells. After 72 h of incubation at 37 °C and 5% CO_2_, the cells were centrifuged at 1000 rpm for 10 min. Pellets were dissolved in 300 µL of complete medium and 30 µL of JC-1 dye was added to each sample. They were incubated for 30 min at 37 °C. Samples were centrifuged at 400 x g for 5 min and washed with assay buffer twice. At the end, 300 µL of assay buffer was added and samples were placed in a black 96-well plate in triplicate. Using a spectrophotometer (Thermo, Varioskan Flash), the samples were measured at 485 and 535 nm for green and at 560 and 595 nm for red fluorescence. The ratios of green/red fluorescence were used to analyze the apoptosis rate.

### Cell Cycle Analysis

Determination of DNA contents of cells was carried out with the help of propidium iodide (PI) dye that specifically binds to DNA. The DNA content decreases in dead cells compared to living cells, so the level of PI gives information about the percentage of cells in the phases of the cell cycle [22]. For this analysis, 1x10^6^ cells per 2 mL were added to 6-well plates and incubated in the absence or presence of increasing concentrations of methylstat at 37 °C and 5% CO_2_. Collected cells were centrifuged at 1200 rpm for 5 min. Supernatants were discarded and PBS was added. In order to fix the cells, cold absolute ethanol (incubated at -20 °C) was added and the cells were incubated at -20 °C deep freeze overnight. The following day, the cells were centrifuged at 1200 rpm for 10 min at 4 °C and washed with cold PBS. Pellets were resuspended with 200 µL of PBS with 0.1% Triton-X 100, and then 20 µL of RNase-A enzyme (200 µg/mL in dH_2_O) was added to each sample and they were incubated at 37 °C for 30 min. At the end of incubation, cells were stained with 20 µL of PI dye (1 µg/mL in dH_2_O) and incubated for 10 min at room temperature. They were analyzed using a flow cytometer.

### cDNA Synthesis and Real-time PCR (RT-PCR)

In order to see the effect of methylstat on the expression levels of anti-apoptotic *BCL2 *and *BCL2L1*, RT-PCR was carried out. First, 1x10^6^ cells/2 mL medium were seeded into 6-well plates and incubated for 72 h at 37 °C and 5% CO_2_. The NucleoSpin RNA kit was used to isolate RNAs according to the manufacturer’s instructions. RNA amounts were measured by NanoDrop (260/280 and 260/230 ratios), and 1000 ng of RNA was used for the synthesis of cDNA. Other components of the PCR mixture were random hexamer primer, buffer, dNTP mix, RNase inhibitor, and reverse transcriptase. The mixtures were incubated at 42 °C for 1 h before incubation at 72 °C for 10 min. These synthesized cDNAs were applied to analyze the changes in expression levels of the *BCL2* and *BCL2L1* genes. The forward primer sequence of the *BCL2* gene was 5’-GCACCTGCACACCTGGAT-3’ and the reverse primer sequence of *BCL2* was 5’-AGCCAGGAGAAATCAAACAGAG-3’, while the forward primer sequence of the *BCL2L1* gene was 5’-AGCCTTGGATCCAGGAGAA-3’ and the reverse primer was 5’-GCTGCATTGTTCCCATAGAGT-3’. Reaction mixtures were prepared to carry out RT-PCR. The utilized Thermo Scientific DyNAmo Flash SYBR^®^ Green qPCR Kit mixtures contain 2X master mix and 50X ROX reference passive dye, and 10 µL of master mix, 5 µL of primer (diluted 1/10), and 5 µL of cDNA (5 ng/µL) were added to Eppendorf tubes for each sample. RT-PCR conditions were adjusted according to the Thermo Scientific DyNAmo Flash SYBR^®^ Green qPCR Kit. Annealing and melting curve temperatures were 60 °C and 40 °C for both the *BCL2 *and *BCL2L1* genes, respectively. The expression level of the *GAPDH* gene was used as an internal positive control. The formula of target gene delta C_t_/reference gene delta C_t_ was used in the calculations. Graphics were plotted with the control sample as 100 and other samples were calculated according to the control sample.

### Statistical Analysis

Statistical analyses and graphs were generated using GraphPad Prism. The statistical significance was detected using one-way analysis of variance (ANOVA) for MTT analysis, annexin, MMP, and caspase-3 enzyme activity and two-way ANOVA for the expression levels of *BCL2* and *BCL2L1* and the MTT analysis of the synergistic effects of methylstat and doxorubicin. A value of p<0.05 was considered to be statistically significant and a value of p<0.001 was considered to be highly statistically significant. Statistics were analyzed using GraphPad Prism 6 for Windows.

## Results

### Cytotoxic Effects of Methylstat on HL-60 Cells

**Figure 1:**
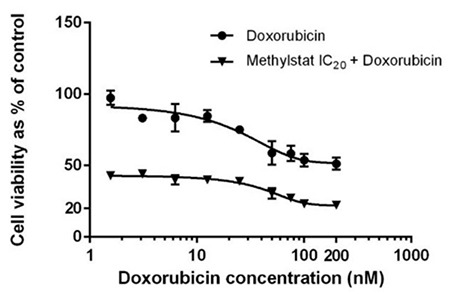
Cytotoxic effects of methylstat on HL-60 acute myeloid leukemia (AML) cells. The IC_50_ value of methylstat is calculated as 1.7 μM by plotted graphs of cell proliferation. Three independent experiments were conducted for data points. The error bars show the standard deviations. Statistical significance was detected by using one-way analysis of variance and p<0.05 was considered to be significant.

Cytotoxic effects of methylstat on HL-60 cells were indicated by MTT cell proliferation assay. There were dose-dependent decreases in the proliferation of HL-60 cells in response to methylstat. The IC_50_ value of methylstat for HL-60 cells was calculated as 1.7 µM for 72 h (Figure 1).

### Methylstat Induced Apoptosis in HL-60 Cells in a Dose-Dependent Manner

**Figure 2:**
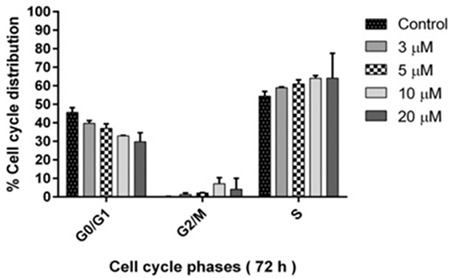
Apoptotic effects of methylstat on HL-60 cells: percentage of dose-dependent apoptotic cell population as compared to untreated cells. The results are the means of 3 independent experiments and the error bars show the standard deviations. Statistical significance was detected by using one-way analysis of variance and p<0.001 was considered to be highly significant.

**Figure 3:**
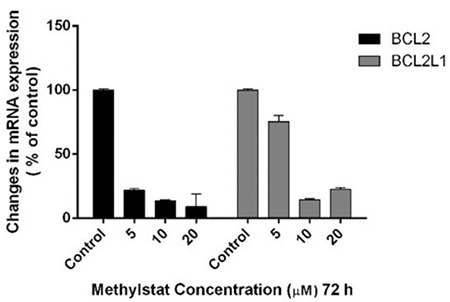
Dot plot diagrams obtained by flow-cytometric analysis of treated HL-60 cells after double staining with annexin V-FITC and PI. Annexin-V FITC-A and PI-A contour plots via quadrant gates show four populations: intact cells in lower-left quadrant, FITC-negative/PI-negative; early apoptotic cells in lower-right quadrant, FITC-positive/PI-negative; late apoptotic or necrotic cells in upper-right quadrant, FITC-positive/PI-positive; necrotic cells in upper-left quadrant, FITC-negative/PI-positive.

Methylstat-induced apoptosis in HL-60 cells was demonstrated by annexin V and PI staining. Changes in apoptotic cell populations by incremental concentrations of methylstat were detected by flow cytometry. There were 1.6-, 10-, 20-, and 22-fold increases in the apoptotic cell population in response to 3, 5, 10, and 20 µM methylstat, respectively, as compared to the untreated control (Figure 2). The contour plots with quadrant gates also indicated that the cell population shifts toward the late apoptotic and early apoptotic quadrants as compared to control cells (which are double-negative on the lower left side of Figure 3).

### Methylstat Stimulates Loss of MMP in HL-60 Cells in a Dose-Dependent Fashion

**Figure 4:**
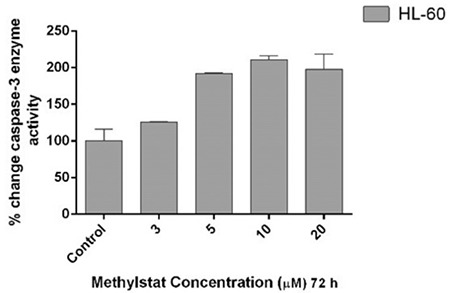
Effects of methylstat on loss of mitochondrial membrane potential. The data are indicated as the means of at least two independent experiments and the error bars show the standard deviations. Statistical significance was detected by using one-way analysis of variance and p<0.001 was considered to be highly significant.

During apoptosis, the loss of MMP is a crucial mediator. Consequently, the loss of MMP for HL-60 cells for the same concentrations of methylstat was detected with the JC-1 Mitochondrial Membrane Potential Assay Kit. Considering the results, the loss of MMP was increased 2.5-, 17-, 20-, and 27-fold in HL-60 cells exposed to 3, 5, 10, and 20 µM methylstat, respectively, with respect to the control group (Figure 4).

### Methylstat Induces Caspase-3 Enzyme Activity in a Dose-Dependent Manner in HL-60 Cells

**Figure 5:**
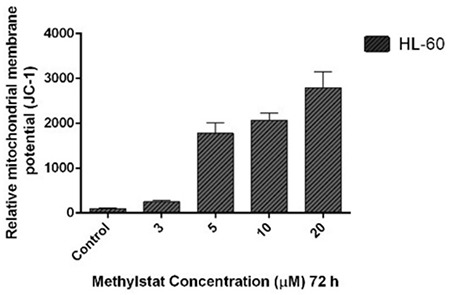
Effects of methylstat on caspase-3 enzyme activity. The data are indicated as the means of at least two independent experiments and the error bars show the standard deviations. Statistical significance was detected by using one-way analysis of variance and p<0.05 was considered to be significant.

In order to examine methylstat-induced apoptosis, caspase-3 enzyme activity was also determined in HL-60 cells exposed to the same concentrations of methylstat using a caspase-3 colorimetric assay kit. There were 1.3-, 1.9-, 2.1-, and 1.9-fold increases of caspase-3 enzyme activity with respect to the untreated control group in HL-60 cells treated with 3, 5, 10, and 20 µM methylstat, respectively (Figure 5).

### Methylstat Induced Apoptosis by Downregulating Expression Levels of the *BCL2* and *BCL2L1* Genes in HL-60 Cells

**Figure 6:**
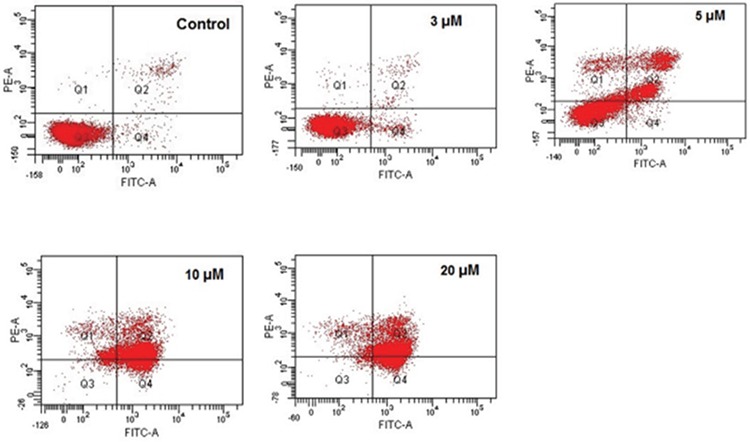
Changes in mRNA levels of anti-apoptotic *BCL2* and *BCL2L1* genes. Statistical significance was detected by using two-way analysis of variance and p<0.001 was considered to be highly significant.

The expression levels of the anti-apoptotic genes *BCL2* and *BCL2L1* for HL-60 cells treated with 5,10, and 20 µM methylstat decreased by 78%, 86%, and 91% and by 24%, 85%, and 77%, respectively, as compared to the control (Figure 6).

### Methylstat Arrested Cell Cycle Progression in G2/M and S Phases in HL-60 Cells

**Figure 7:**
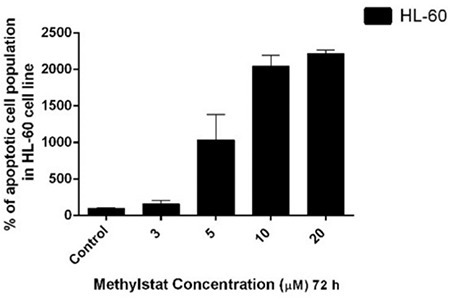
Effects of methylstat on cell cycle progression of HL-60 cells. Three independent experiments were conducted for data points.

The cytostatic effects of methylstat were displayed by DNase-free RNase and PI staining using flow cytometry. As shown in Figure 7, methylstat-treated HL-60 cells have increased cell populations in the S and G2/M phases as the concentration increases, while the percentage of cells arrested at the G0/G1 phase decreases. These results indicate that methylstat arrests the cell division cycle in the S and G2/M phases.

### Synergistic Effects of Methylstat and Doxorubicin on HL-60 Cells

**Figure 8:**
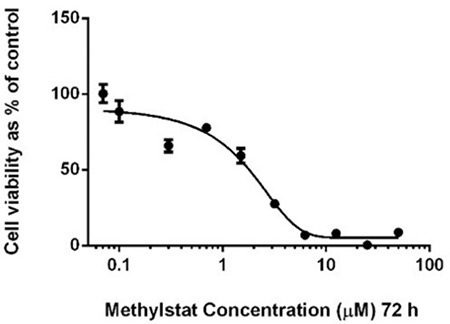
Synergistic cytotoxic effects of doxorubicin in combination with methylstat (72 h). The results are the means of 4 independent experiments and error bars indicate the standard deviations. Statistical significance was detected by using one-way analysis of variance and p<0.001 was considered to be highly significant.

Doxorubicin is known as a common treatment option for AML. We examined the possible synergistic effect of the combination of doxorubicin and methylstat on HL-60 cells. With this aim, HL-60 cells were exposed to increasing concentrations of doxorubicin together with IC_20_ concentrations of methylstat. There were significant synergistic effects of this combination as compared to either agent alone (Figure 8).

## Discussion

The importance of epigenetic modifications increases with large-scale research. Histone-modifying enzymes are the subject of special interest because they are main players in epigenetics. Histone methylation and demethylation play important roles in diverse pathological and biological events, including cancers. The reversal of histone demethylations can be a potential treatment strategy for cancer due to the overexpression of histone demethylases in various cancer types [23].

Over the last decade, many studies have focused on the JmjC family of histone demethylases. A number of researchers have indicated the association between JHDMs and several physiological and pathological circumstances including cancer, inflammation, development, metabolism, and neurological disorders. The relevance of dysregulation of JHDMs in various types of cancer makes them a potential candidate to target. If abnormal JmjC demethylase activity is modulated, it can lead to normal transcriptional arrangements. Therefore, JmjC domain inhibitors might have therapeutic potentials for the treatment of cancer [24,25,26,27].

Here, we addressed the particular question of whether a selectively active inhibitor of JHDMs has anti-proliferative effects. There is no significant knowledge about the relevance of JHDMs for AML. In this study, we aimed to detect the cytotoxic, cytostatic, and apoptotic effects of methylstat on HL-60 acute pre-myelocytic leukemia cells. The cells were treated with increasing concentrations of methylstat. Methylstat showed cytotoxic/antiproliferative effects in a dose-dependent manner. To determine the apoptotic effects of methylstat, annexin FITC/PI double staining, caspase-3 enzyme activity, and the loss of MMP were studied (72 h). With respect to these apoptotic assays, methylstat exhibited a dose-dependent induction of apoptosis and increased the percentage of apoptotic cells as compared to untreated controls. The mRNA expression levels of the foremost anti-apoptotic genes, *BCL2* and *BCL2L1*, were detected by RT-PCR. The results supported the previous apoptotic assays and indicated that *BCL2* and *BCL2L1* expressions were decreased with incremental concentrations of methylstat. Besides the induction of apoptosis, cytostatic effects were checked to see how methylstat mediates cell cycle arrest in HL-60 cells. Incubation of HL-60 cells for 72 h with methylstat led to cell cycle arrest at the G2/M and S phases. On the other hand, to increase the therapeutic effects and to reduce the side effects, it might be better to combine conventional therapies with epigenetic modifiers such as demethylase inhibitors. Therefore, in our study, we combined methylstat with the most common chemotherapy agent, doxorubicin, in order to see whether they have synergistic effects. Doxorubicin had 2 times more anti-proliferative effect together with an IC_20_ value of methylstat on HL-60 cells.

## Conclusion

According to our results, methylstat has potent apoptotic effects on HL-60 cells. It might have therapeutic potential to treat AML through the induction of apoptosis and anti-proliferative effects. However, it would be more effective to show the chemotherapy response of methylstat with more cell lines and on mouse models of human AML. That could be more helpful for a better understanding of the clinical implications of methylstat.

Chemotherapy targets both healthy, normal cells and cancer cells. Therefore, it is important for an inhibitor to be specific for cancer cells in order to minimize the side effects. For further investigations, it might be better to apply methylstat to human leukocyte cultures, expecting a higher IC_50_ value compared to AML cell lines. Furthermore, mice xenografts might be helpful to comprehend the possible potential of methylstat for clinical implications. In addition, there are a number of JmjC type subfamilies and it would help to understand which of them are overexpressed specifically for AML. Depending on that, new specific therapeutic inhibitors for these JmjC demethylases might be designed and applied in vivo and in vitro.
